# Validity and Reliability of the Healthy Aging Instrument in Older Adults With and Without Fear of Falling

**DOI:** 10.1093/geroni/igaa057.2956

**Published:** 2020-12-16

**Authors:** Ladda Thiamwong

**Affiliations:** College of Nursing, University of Central Florida, Orlando, Florida, United States

## Abstract

An instrument to measure healthy aging has not been validated in older adults with and without fear of falling, expressing a methodological gap that limits research in this field. This cross-sectional study aimed to validate the 35-item Healthy Aging Instrument (HAI) in adults aged 60 years and over. 389 community-dwelling older adults from two provinces in Thailand were recruited. The mean age of participants was 70.4 years, and 63% were women. The HAI showed good construct validity and internal reliability in the total group and subgroups with respect to levels of fear of falling. All correlations among the HAI, Fall Risk Assessment Test, Fall Efficacy Scale-International, and full tandem stance test were significant. While Western cultures value the expression of thoughts and feelings, it is not particularly encouraged in Asian cultures. The HAI demonstrates good psychometric properties and provides the basis for measuring healthy aging in Asian older adults.

